# An Untargeted Metabolomics Investigation of Milk from Dairy Cows with Clinical Mastitis by ^1^H-NMR

**DOI:** 10.3390/foods10081707

**Published:** 2021-07-23

**Authors:** Chenglin Zhu, Kaiwei Tang, Xuan Lu, Junni Tang, Luca Laghi

**Affiliations:** 1 College of Food Science and Technology, Southwest Minzu University, Chengdu 610041, China; chenglin.zhu@swun.edu.cn (C.Z.); tangkaiwei@stu.swun.edu.cn (K.T.); luxuan@stu.swun.edu.cn (X.L.); 2 Department of Agro-Food Science and Technology, University of Bologna, 47521 Cesena, Italy; l.laghi@unibo.it

**Keywords:** milk, mastitis, metabolome, ^1^H-NMR, pathway analysis

## Abstract

Mastitis is one of the diseases with the highest incidence in dairy cows, causing huge economic losses to the dairy industry all over the world. The aim of the study was to characterize mastitic milk metabolome through untargeted nuclear magnetic resonance spectroscopy (^1^H-NMR). Taking advantage of the high reproducibility of ^1^H-NMR, we had the opportunity to provide quantitative information for all the metabolites identified. Fifty-four molecules were characterized, sorted mainly into the chemical groups, namely amino acids, peptides and analogues, carbohydrates and derivates, organic acids and derivates, nucleosides, nucleotides and analogues. Combined with serum metabolomic investigations, several pathways were addressed to explain the mechanisms of milk metabolome variation affected by clinical mastitis, such as tricarboxylic acid cycle (TCA cycle) and phenylalanine, tyrosine and tryptophan biosynthesis. These results provide a further understanding of milk metabolome altered by clinical mastitis, which can be used as a reference for the further milk metabolome investigations.

## 1. Introduction

As one of the diseases with the highest incidence and adverse consequences in dairy industry, bovine mastitis has been evaluated continuously from the last century [1]. The negative consequences include the reduction of milk yield and quality, increased treatment costs and cow mortality. Even worse, bovine mastitis is also considered as the major cause of antibiotics overuse in dairy farms, with many potential adverse effects on consumers [2]. In practice, the California mastitis test (CMT) is well known as a rapid and convenient method to estimate milk somatic cell count (SCC) in mastitis diagnosis at farm level [3]. Inconveniently, noninfectious factors (lactation stage, parity, season, milking frequency) can have the opportunity to affect SCC [4]. Therefore, additional diagnosis methods would be desirable to elevate the accuracy of bovine mastitis detection.

Metabolomics, being downstream of genomics, transcriptomics and/or proteomics, is regarded as the most comprehensive representation of an organism’ phenotype [5]. The key feature of metabolomics of a biofluid is that it seeks to provide qualitative and quantitative information of all its low weight metabolites (<900 Da). This characterization could provide holistic information of a system through untargeted mode, so that it could be ideal in the study of the biochemical responses to intramammary stimuli [5]. Among all the metabolomic platforms, ^1^H-NMR spectroscopy is one of the most commonly used, because the limited sample preparation, its non-invasive nature and highly reproducible quantifications counterbalance a sensitivity lower than mass spectrometry platforms. Hereby, ^1^H-NMR spectroscopy has been applied in domestic animals to obtain metabolites’ profiles of a few number of biofluids, among which urine [6], plasma [7], serum [8] and tracheal wash [9].

Currently, more and more researchers put their eyes on how the metabolome is altered by multiple pathological conditions. In terms of bovine mastitis, several remarkable studies have described how the milk metabolome is altered by mastitis linked to live bacterial pathogens. Sundekilde et al. revealed that the concentrations of isoleucine, lactate, 3-hydroxybutyrate, butyrate and acetate were increased, whereas fumarate and hippurate concentrations were declined in milk with high numbers of somatic cells [10]. Xi et al. found that less concentrations of d-glycerol-1-phosphate, *sn*-glycero-3-phosphocholine, glucose, citrate, 4-hydroxyphenyllactate, hippurate and carnitine were found in the clinical mastitic milk samples [11].

Although previous studies have pointed out several potential biomarkers in milk samples from dairy cows with mastitis, there is to our best knowledge limited information presently about the mechanism through which milk metabolome is altered by mastitis. Moreover, there is limited information about expected concentrations of metabolites from mastitic milks. To address these issues, in the present work ^1^H-NMR based metabolomic investigations and pathway analysis are applied to gain more information about milk metabolome alterations caused by mastitis. A related study has proved that serum metabolome can be linked to milk metabolomic phenotype and assist in better understanding mastitis intervention [12]. Therefore, in order to observe the mechanism of milk metabolome affected by mastitis comprehensively, in the current study serum samples from the same cows were also involved into the metabolomic investigations.

## 2. Materials and Methods 

### 2.1. Sampling

The experimental designs and protocols were all approved by the Animal Ethics Committee of Southwest Minzu University (Chengdu, China) and followed the recommendations of the academy’s guidelines for animal research. Ten Chinese Holstein cows (parity: 2–4, days in milk: 150–195 d) were involved in the study, five healthy and five with clinical mastitis. In the current study, the cows with clinical mastitis were comprehensively judged by experienced veterinary surgeons, based on the clinical manifestations of the udder (fever, redness and swelling) and the result of CMT. The samples from the cows with mastitis were collected in routine physical examinations at a pasture in Chengdu, Sichuan. Milk samples from healthy dairy cows were collected during the routine milking process by an automatic milking rotary system (Ruishengyuan Machinery Assembly Co., Ltd., Hengshui, Hebei, China). Venous blood samples were collected by syringe by an experienced vet.

All involved cows were housed in free housing systems, fed with total mixed rations (TMR) in accordance with standard practices during their indoor period and milked twice daily in the same pasture. Immediately after milk and blood samples collection, all the samples were refrigerated with ice for transport to the laboratory within two hours.

### 2.2. Metabolome Analysis

In order to fulfill the requirements of ^1^H-NMR analysis, 0.7 mL of milk samples were added to 0.8 mL CHCl_3_, and then vortex mixing for 3 min and centrifuging for 15 min at 18,630 g and 4 °C, in order to remove fat from samples. 0.5 mL of the supernatants were added to 0.2 mL of a D_2_O solution of 3-(trimethylsilyl)-propionic-2,2,3,3-d_4_ acid sodium salt (TSP) 10 mmol/L, used as NMR chemical-shift reference, buffered at pH 7.00 ± 0.02 by means of 1 mol/L phosphate buffer. In order to avoid microbial proliferation, 10 μL of NaN_3_ 2 mmol/L was also added. Finally, each sample was centrifuged again at the above conditions. Serum samples were prepared for ^1^H-NMR analysis according to Zhu et al. [13].

^1^H-NMR spectra were acquired at 298 K with an AVANCE III spectrometer (Bruker, Milan, Italy) operating at a frequency of 600.13 MHz. The signals from broad resonances originating from large molecules were suppressed by a CPMG-filter composed by 400 echoes with a τ of 400 μs and a 180° pulse of 24 μs, for a total filter of 330 ms. The HOD residual signal was suppressed by means of pre-saturation. This was done by employing the cpmgpr1d sequence, part of the standard pulse sequence library. Each spectrum was acquired by summing up 256 transients using 32 K data points over a 7184 Hz spectral window, with an acquisition time of 2.28 s. 

^1^H-NMR spectra phase was manually adjusted in Topspin, following the subsequent adjustments which were performed in in-house R computational language scripts. After removing the residual water signal, the spectra were baseline adjusted by means of peak detection according to the “rolling ball” principle [14] implemented in the baseline R package [15]. Differences in water and protein content among samples were taken into consideration by probabilistic quotient normalization [16] applied to the entire spectra array. The signals were assigned by comparing their chemical shift and multiplicity with Chenomx software library (Chenomx Inc., Edmonton, NA, Canada, ver 8.4). 

In order to apply NMR as a quantitative technique, the recycle delay was set to 5 s, keeping into consideration the relaxation time of the protons under investigation. Integration of the signals was performed for each molecule by means of rectangular integration.

### 2.3. Statistical Analysis

Statistical analysis was conducted in R computational language [17]. Molecules whose concentration altered between groups were looked for by means of Student *t*-test. For the purpose, *p*-value below 0.05 was accepted. Prior to analysis, variables that were not-normally distributed were transformed according to Box and Cox [18].

To overview the trends underlying the metabolome of the samples, we setup a robust principal component analysis (rPCA) model [19], on the molecules accepted by the above described univariate analysis. For the model, we calculated the score-plot, and the projection of the samples in the PC space, tailored to highlight the structure of the data. Moreover, Pearson correlation plot was calculated, relating each molecule’s concentration to the components of the model.

Pathway analysis was applied by using MetaboAnalyst 5.0 [20]. By combining the results obtained from the pathway enrichment analysis, the most relevant pathways involved were identified based on the *Bos taurus* KEGG pathway library [21]. For the purpose, only molecules accepted by the above univariate analysis were considered. 

## 3. Results

### 3.1. ^1^H-NMR Spectra of Milk Samples

**Figure 1:**
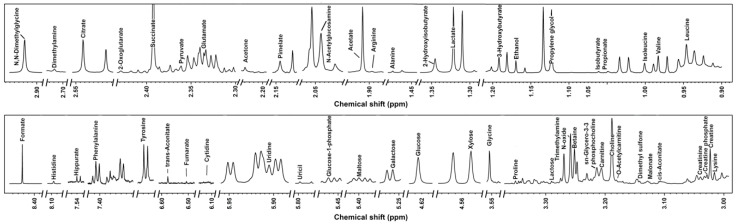
^1^H-NMR spectrum from one milk sample representative of all the registered spectra. The name of each molecule appears over the signal used for its quantification. To ease the reader’s visual inspection, for each portion a spectrum with a convenient signal-to-noise ratio has been selected.

Signals from ^1^H-NMR were assigned as pictorially described in Figure 1, while molecules’ concentrations and *p* values obtained from t-test are reported in Table 1. To ease the readers’ reproduction of our results, the functional groups and ppm for each identified metabolite are reported in Table S1. A total of 54 molecules was characterized, which could be sorted mainly into several chemical groups, namely amino acids, peptides and analogues, carbohydrates and derivates, organic acids and derivates, nucleosides, nucleotides and analogues.

### 3.2. Milk Metabolome Affected by Clinical Mastitis

**Figure 2:**
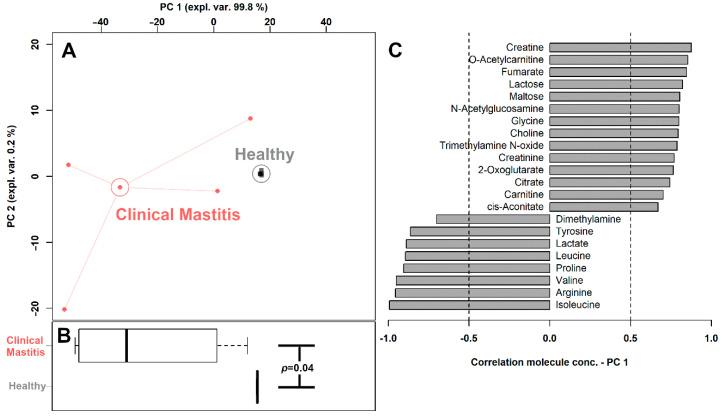
rPCA model built on the concentration of the molecules which showed a statistically significant difference between the two groups. In the score-plot (**A**), samples from the two groups are represented with squares (Healthy) and circles (Clinical Mastitis). The wide, empty circles represent the median of each samples’ group. The position of the samples along PC1 is summarized in the boxplot (**B)**. The loading plot (**C**) reports the significant correlations (*p* < 0.05) between the concentration of each substance and its importance over PC 1.

Twenty-two of the quantified molecules were significantly different between the two groups, namely creatine, O-acetyl-carnitine, fumarate, lactose, maltose, N-acetylglucosamine, glycine, choline, trimethylamine N-oxide, creatinine, 2-oxoglutarate, citrate, 2-oxoglutarate, citrate, carnitine, *cis*-aconitate, dimethylamine, tyrosine, lactate, leucine, proline, valine, arginine and isoleucine. In order to view an overall trend of the so characterized molecules, their concentrations were employed as a basis for an rPCA model, shown in Figure 2.

The PC 1 of its score-plot (Figure 2A), accounting for as much as 99.8% of the entire samples’ variability represented by the model, nicely summarizes the differences between the samples from the two groups. In detail, milk from clinical mastitis cows was found mainly to be characterized by higher concentrations of dimethylamine, tyrosine, lactate, leucine, proline, valine, arginine and isoleucine and lower concentrations of creatine, O-acetyl-carnitine, fumarate, lactose, maltose, N-acetylglucosamine, glycine, choline, trimethylamine N-oxide, creatinine, 2-oxoglutarate, citrate, 2-oxoglutarate, citrate, carnitine and *cis*-aconitate. 

**Figure 3:**
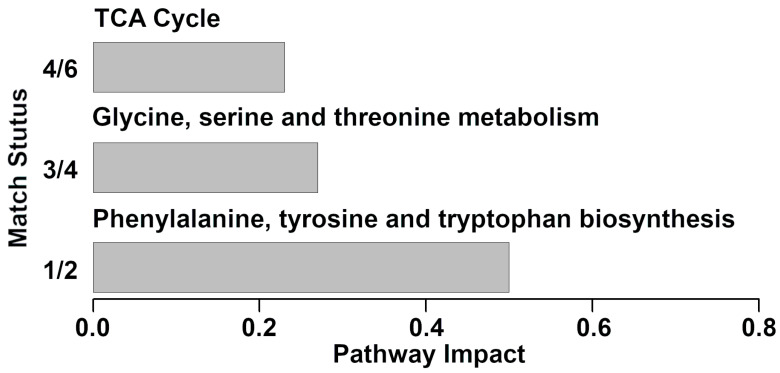
Metabolic pathways evidenced by enrichment analysis performed on the milk’s metabolites significantly differently between the two groups (impact value > 0.2).

The 22 molecules were used as a basis for a pathway enrichment analysis, to identify the most relevant pathways differentiating the groups. Three pathways were highlighted, namely phenylalanine, tyrosine and tryptophan biosynthesis, glycine, serine and threonine metabolism and TCA cycle (Figure 3).

### 3.3. Pathway Analysis of Serum Metabolites Affected by Clinical Mastitis

**Figure 4:**
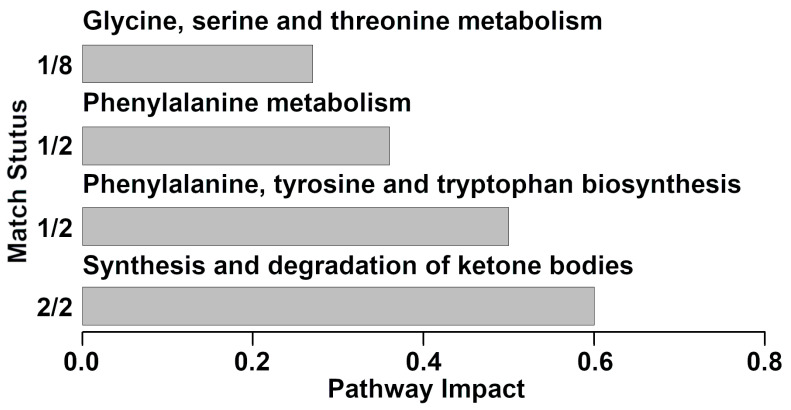
Metabolic pathways evidenced by enrichment analysis based on the statistically significant metabolites quantified from serum samples between different groups (impact value > 0.2).

In serum samples, nine molecules of the 54 quantified showed significant differences between the two groups (data are not shown). The nine molecules were used as a basis for a pathway enrichment analysis, to identify the most relevant pathways differentiating the groups. Four pathways were highlighted (Figure 4), namely synthesis and degradation of ketone bodies, phenylalanine, tyrosine and tryptophan biosynthesis, phenylalanine metabolism and glycine, serine and threonine metabolism.

## 4. Discussion

As one of the most economically important diseases in the dairy production industry, bovine mastitis has been studied from a variety of perspectives, such as milk yield and composition [22], milk bacterial communities [23] and milk metabolome [24]. In terms of milk metabolome, there are no complete reports containing quantitative information for each metabolite that can be identified by ^1^H-NMR. Moreover, the mechanisms through which milk metabolome is affected by mastitis have still been only partially elucidated. In order to fill these gaps, the present work firstly attempts to give reference concentrations of the molecules represented most commonly in the milk metabolome of dairy cows with mastitis, as observable by ^1^H-NMR. A total of 54 metabolites were unambiguously identified and quantified, a number higher than those previously obtained based on the same platform [3,10,25]. Serum metabolome of the same cows with/without mastitis was observed in parallel, granting a multifaceted observation of the underlying mechanisms relating cows’ metabolome to mastitis. A potential limit of the work could be represented by the limited number of samples analyzed, even if such number is in line with previous works devoted to the evaluation of the consequences of mastitis on milk metabolome [22,26]. In this respect, it is worth noticing that, despite the large inter-variability among samples evidenced in Figure 2, none of the samples could be considered as an outlier.

Compared to healthy animals, the concentrations of eight molecules appeared significantly elevated in cows with clinical mastitis, namely dimethylamine, tyrosine, lactate, leucine, proline, valine, arginine and isoleucine. Interestingly, six of the eight molecules belonged to the chemical group of amino acids. Such finding is in line with previous studies, reporting that the concentrations of free amino acids (i.e., arginine, valine, isoleucine and proline) increase in milk samples with clinical mastitis [10,27]. Statistically significant increases in milk amino acids might be related to the enhancement of pathogen-specific fermentative processes and to protein degradation activities [28]. The results of pathway analysis of milk and serum seem to confirm the above phenomenon. Among all the underlining pathways, it is worthy to notice that phenylalanine, tyrosine and tryptophan biosynthesis was addressed as one of the main pathways. Focusing on phenylalanine, even though we failed to find any significant differences in milk samples, we were able to evidence these in serum. Phenylalanine is an essential amino acid which can be transformed into tyrosine through the action of phenylalanine hydroxylase and a biopterin cofactor [29]. This molecule can also be regarded as the precursor of catecholamines, neurotransmitters and adrenaline-like substances [30]. As the main component of many proteins, peptides, and even enkephalins, tyrosine is an important amino acid and is also the precursor for hormones such as catechol-estrogens and thyroxin [31].

Lactate is the major end product of carbohydrates’ metabolism and can be produced by microorganisms in milk or by anaerobic epithelial respiration in oxygen-deprived conditions following mastitis [32]. It has been shown that the presence of bacteria in the milk leads to a distinct metabolic fingerprint, characterized by high levels of lactate [33]. In addition, the fold change of lactate was in agreement with previous reports, at around 30-fold [33]. Metabolites present in milk can originate from a range of different sources, including the transfer from blood, the active secretion or leakage from damaged somatic cells, bacteria living in the milk, or secreted from the mammary epithelial cells. Therefore, it is of help to combine observations from different biofluids, such as serum, into a single metabolomic investigation so to clarify the mechanisms linking milk metabolome to clinical mastitis.

Several molecules showed a decreasing trend from healthy to mastitis affected cows. These molecules mainly gave information about energy generation (fumarate, lactose, maltose, 2-oxoglutarate, citrate and *cis*-aconitate), protein digestion (creatine, O-acetyl-carnitine, glycine, creatinine and carnitine) or diet (choline N-acetylglucosamine, and trimethylamine N-oxide). Such results were in line with previous studies. For instance, Thomas et al. reported that carbohydrates and energy related metabolites (i.e., citrate, *cis*-aconitate and 2-oxoglutarate) were less concentrated in the clinical mastitis milks [34]. The pathway analyses conducted for the present investigation confirmed this phenomenon, with TCA cycle pathway highlighted in milk, and synthesis and degradation of ketone bodies pathway highlighted in serum. The TCA cycle is the basic metabolic pathway in mitochondria and is critically important for dairy cows’ health. The variation in some of its metabolites is likely to be a combination of two aspects. On one side, the pathogens are expected to divert these for their metabolic activities and growth, along with mastitis progresses. On the other side, the TCA cycle in dairy cows with mastitis could be downregulated. Taken together, our results shed light on the inference that clinical mastitis modifies the metabolite balance in cow milk by disturbing the TCA cycle in the mammary gland. N-acetylglucosamine, a derivative amide of glucose and a secondary amide between glucosamine and acetic acid, is derived from amino sugars metabolism [35]. Fumarate is an intermediate of the tricarboxylic acid cycle and urea cycle. The decrease in fumarate in milk samples with clinical mastitis may be explained by changes in the energy metabolism in the cow due to a bacterial infection or ketosis leading to impaired tricarboxylic acid function, which in turn results in active secretion or leakage of fumarate from the mammary epithelial cells into the milk [10].

Carnitine metabolism is another metabolic pathway that was downregulated during clinical mastitis. Carnitine is responsible for the transportation of long-chain fatty acids from the cytosol to the mitochondrial matrix and, in turn, further regulates the energy metabolism [36]. O-acetyl-carnitine plays an important role in β-oxidation of free fatty acid [37]. With the vital functions of carnitine and O-acetyl-carnitine as mediators in transporting long-chain fatty acid into the mitochondria for β-oxidation, the decreases in carnitine in clinical mastitic milk samples may suggest a hampered energy metabolism in bovine mastitis. Glycine is the predominant amino acid for creatine synthesis in the liver [38]. As an intermediate metabolite in energy reactions, creatine plays an important role in regulating negative energy balance in dairy cows and could also be considered as a potential diagnostic biomarker for heat stress [39]. Creatinine is synthesized, linked to the absorption of creatine phosphate by the muscles, then released to the serum and cleared by kidneys [13]. In dairy cattle, this molecule has been found to be proportional to muscle activity, with specific reference to respiration rates and heart [40]. Lactose and citrate are secreted by the mammary epithelial cells by exocytosis [41]. In addition, several of the molecules we underlined may be involved in the coagulation process in milk, for instance, choline, carnitine, citrate and lactose, hereby affecting the quality, yield and composition of milk [36]. The content of lactose is decreased in milk from ruminants with inflammation of the mammary glands. As dairy cows are affected by clinical mastitis, the mammary cell membrane is damaged, leading to blood constituents which flow into the milk [42]. To keep the osmotic pressure constant, lactose is decreased accordingly. 

## 5. Conclusions

To the best of our knowledge, this is the first work devoted to providing quantitative information about dairy cow milk metabolome with/without mastitis by means of untargeted ^1^H-NMR. A number of metabolites greater than previously reported for milk was characterized, even if the low number of samples analyzed limited the significance of the differences highlighted between cows with/without mastitis. Combined with serum metabolomic investigations, the mechanisms of milk metabolome caused by clinical mastitis have been further clarified. The present study could serve as a reference for further milk metabolome studies. Moreover, it also sheds light on the advantages of the application of multi-biofluid metabolomic investigations.

## Figures and Tables

**Table 1:** Concentrations (mmol/L, median (IQR)) of molecules affected by clinical mastitis in milk samples.

	Healthy	Clinical Mastitis	*p* Value	Trend
Amino Acids, Peptides and Analogues				
Alanine	5.31 × 10^−2^ (1.23 × 10^−2^)	1.68 × 10^−1^ (1.04 × 10^−1^)	0.2411	↑
Arginine	2.30 × 10^−2^ (4.72 × 10^−3^)	2.04 × 10^−1^ (1.13 × 10^−1^)	0.0190	↑
Betaine	4.64 × 10^−2^ (8.52 × 10^−4^)	4.57 × 10^−2^ (4.06 × 10^−2^)	0.9821	↓
Creatine	8.35 × 10^−1^ (7.90 × 10^−2^)	2.06 × 10^−1^ (7.82 × 10^−2^)	0.0106	↓
Creatine phosphate	1.13 × 10^−1^ (7.85 × 10^−2^)	4.49 × 10^−2^ (2.36 × 10^−2^)	0.0589	↓
Creatinine	1.12 × 10^−1^ (1.44 × 10^−2^)	2.39 × 10^−2^ (1.45 × 10^−2^)	0.0000	↓
Glutamate	7.46 × 10^−1^ (3.93 × 10^−1^)	6.57 × 10^−1^ (6.59 × 10^−1^)	0.6295	↓
Glycine	1.63 (1.37)	2.65 (2.49)	0.0000	↑
Histidine	9.35 × 10^−2^ (1.60 × 10^−2^)	2.88 × 10^−2^ (5.00 × 10^−2^)	0.0278	↓
Isoleucine	4.54 × 10^−3^ (3.99 × 10^−3^)	8.45 × 10^−2^ (7.13 × 10^−2^)	0.0021	↑
Leucine	1.10 × 10^−1^ (2.69 × 10^−2^)	5.27 × 10^−1^ (3.93 × 10^−2^)	0.0003	↑
Lysine	1.58 × 10^−1^ (1.61 × 10^−2^)	2.68 × 10^−1^ (1.86 × 10^−1^)	0.1177	↑
*N*,*N*-Dimethylglycine	3.16 × 10^−3^ (1.42 × 10^−4^)	3.06 × 10^−3^ (1.40 × 10^−2^)	0.9092	↓
Phenylalanine	2.14 × 10^−2^ (7.10 × 10^−3^)	6.47 × 10^−2^ (6.65 × 10^−2^)	0.2731	↑
Proline	2.46 × 10^−3^ (2.21 × 10^−3^)	8.15 × 10^−2^ (8.95 × 10^−2^)	0.0028	↑
Tyrosine	3.90 × 10^2^ (1.48 × 10^−3^)	8.57 × 10^−2^ (4.20 × 10^−2^)	0.0214	↑
Valine	1.77 × 10^−2^ (6.67 × 10^−3^)	1.46 × 10^−1^ (1.18 × 10^−1^)	0.0017	↑
Carbohydrates and Derivates				
Acetone	9.82 × 10^−3^ (1.27 × 10^−3^)	4.81 × 10^−3^ (6.18 × 10^−3^)	0.0728	↓
Galactose	7.32 × 10^−1^ (1.91 × 10^−1^)	2.71 × 10^−1^ (3.71 × 10^−1^)	0.3737	↓
Glucose	6.77 × 10^−1^ (2.96 × 10^−2^)	1.88 × 10^−1^ (2.53 × 10^−1^)	0.1906	↓
Glucose-1-phosphate	1.58 × 10^−1^ (7.73 × 10^−2^)	1.05 × 10^−2^ (7.54 × 10^−3^)	0.0675	↓
Lactose	119 (8.77)	15.8 (23.3)	0.0012	↓
Maltose	1.25 × 10^−1^ (7.10 × 10^−2^)	4.08 × 10^−3^ (3.78 × 10^−3^)	0.0121	↓
Xylose	1.73 (4.06 × 10^−1^)	5.43 × 10^−1^ (4.60 × 10^−1^)	0.1879	↓
Organic Acids and Derivates				
2-Hydroxyisobutyrate	3.34 × 10^−3^ (8.25 × 10^−4^)	2.12 × 10^−2^ (8.16 × 10^−1^)	0.2667	↑
2-Oxoglutarate	2.46 × 10^−1^ (6.12 × 10^−2^)	3.46 × 10^−2^ (1.82 × 10^−2^)	0.0000	↓
3-Hydroxybutyrate	9.40 × 10^−2^ (2.11 × 10^−2^)	1.83 × 10^−1^ (1.14 × 10^−1^)	0.0012	↑
Acetate	3.68 × 10^−2^ (9.56 × 10^−3^)	3.91 × 10^−1^ (4.18 × 10^−1^)	0.1093	↑
Citrate	8.92 (1.32)	1.63 (1.02)	0.0002	↓
Formate	3.19 × 10^−2^ (5.96 × 10^−3^)	6.40 × 10^−2^ (2.47 × 10^−1^)	0.4165	↑
Fumarate	2.8910^−2^ (3.40 × 10^−4^)	6.9710^−3^ (4.40 × 10^−3^)	0.0002	↑
Hippurate	5.76 × 10^−2^ (3.27 × 10^−2^)	4.03 × 10^−2^ (3.78 × 10^−2^)	0.0517	↓
Iso-butyrate	2.57 × 10^−3^ (8.42 × 10^−4^)	5.77 × 10^−3^ (3.84 × 10^−3^)	0.1710	↑
Lactate	7.84 × 10^−2^ (2.50 × 10^−2^)	3.10 (5.14)	0.0129	↑
Malonate	1.35 × 10^−2^ (1.22 × 10^−2^)	5.62 × 10^−3^ (3.09 × 10^−3^)	0.0644	↓
Pimelate	1.56 × 10^−1^ (2.48 × 10^−3^)	4.80 × 10^−2^ (2.35 × 10^−2^)	0.1168	↓
Propionate	2.01 × 10^−3^ (3.68 × 10^−4^)	4.35 × 10^−4^ (1.08 × 10^−4^)	0.1625	↓
Pyruvate	2.01 × 10^−2^ (4.79 × 10^−3^)	3.09 × 10^−2^ (1.39 × ^10−2^)	0.0128	↑
Succinate	2.97 × 10^−2^ (4.64 × 10^−3^)	3.26 × 10^−2^ (1.13 × 10^−1^)	0.6861	↑
Nucleosides, Nucleotides and Analogues				
Cytidine	2.95 × 10^−2^ (1.54 × 10^−3^)	9.68 × 10^−3^ (1.51 × 10^−3^)	0.0005	↓
Dimethylamine	2.42 × 10^−3^ (4.04 × 10^−4^)	6.86 × 10^−3^ (9.24 × 10^−3^)	0.0414	↑
N-Acetylglucosamine	9.22 × 10^−1^ (4.77 × 10^−1^)	4.97 × 10^−1^ (3.64 × 10^−1^)	0.0311	↓
Trimethylamine-N-oxide	1.69 × 10^−1^ (6.86 × 10^−3^)	4.96 × 10^−2^ (2.54 × 10^−2^)	0.0002	↓
Uridine	2.94 × 10^−2^ (1.35 × 10^−2^)	2.92 × 10^−2^ (4.66 × 10^−1^)	0.6721	↓
Miscellaneous				
Carnitine	2.60 × 10^−1^ (1.29 × 10^−1^)	1.43 × 10^−2^ (2.21 × 10^−2^)	0.0485	↓
Choline	3.37 × 10^−1^ (3.82 × 10^−2^)	7.41 × 10^−2^ (3.43 × 10^−2^)	0.0009	↑
*cis*-Aconitate	1.52 × 10^−1^ (1.69 × 10^−2^)	3.46 × 10^−2^ (2.18 × 10^−2^)	0.0000	↓
Dimethyl sulfone	1.73 × 10^−2^ (9.08 × 10^−3^)	7.56 × 10^−3^ (4.14 × 10^−3^)	0.0035	↓
Ethanol	4.37 × 10^−2^ (3.45 × 10^−3^)	7.59 × 10^−2^ (8.97 × 10^−2^)	0.5625	↑
O-Acetyl-carnitine	5.54 × 10^−2^ (6.16 × 10^−3^)	1.48 × 10^−2^ (1.14 × 10^−2^)	0.0451	↓
Propylene glycol	1.03 × 10^−2^ (2.09 × 10^−3^)	2.76 × 10^−2^ (4.99 × 10^−1^)	0.1390	↑
*trans*-Aconitate	2.31 × 10^−2^ (1.97 × 10^−3^)	1.25 × 10^−2^ (6.68 × 10^−3^)	0.1530	↓
*sn*-Glycero-3-phosphocholine	1.23 (1.48 × 10^−1^)	2.30 × 10^−1^ (1.20 × 10^−1^)	0.0799	↓
Uricil	1.35 × 10^−2^ (2.23 × 10^−3^)	1.60 × 10^−2^ (1.49 × 10^−2^)	0.7987	↑

## Data Availability

All data generated or analyzed during this study are included in this published article and its supplementary information file.

## Supplementary Materials

The following are available online at https://www.mdpi.com/article/10.3390/foods10081707/s1, Table S1: Information for molecules identification by means of 1H-NMR.
