# Supplementary material for: An Untargeted Metabolomics Investigation of Milk from Dairy Cows with Clinical Mastitis by 1H-NMR
Source: Foods. 2021 Jul 23;10(8):1707. doi: 10.3390/foods10081707 (PMC8394248; doi:10.3390/foods10081707)
Supplement: Supplementary file 1 [file foods-10-01707-s001.zip › foods-1303538-supplementary.pdf]

An untargeted metabolomics investigation of Milk from Dairy Cows with Clinical Mastitis by ¹H-NMR

Chenglin Zhu[1], Kaiwei Tang[1], Xuan Lu[1], Junni Tang[1,*] and Luca Laghi[2]

Table S1 Information for molecules identification by means of ^{1}H-NMR

	Functional Group	Multiplicity	ppm
2-Hydroxyisobutyrate	CH_3	s	1.3466
2-Oxoglutarate	CH_2-4	t	2.4279
3-Hydroxybutyrate	CH_3	d	1.1917
Acetate	CH_3	s	1.9071
Acetone	CH_3	s	2.2199
Alanine	CH_3	d	1.4756
Arginine	CH_2-3	m	1.8964
Betaine	CH_3	s	3.2511
Carnitine	CH_3	s	3.2040
Choline	CH_3	s	3.1889
cis-Aconitate	CH_2	d	3.1107
Citrate	CH_2	d	2.5344
Creatine	CH_3	s	3.0231
Creatine phosphate	CH_3	s	3.0302
Creatinine	CH_3	s	3.0357
Cytidine	CH-6	d	6.1161
Dimethyl sulfone	CH_3	s	3.1418
Dimethylamine	CH_3	s	2.7100
Ethanol	CH_3	t	1.1692
Formate	CH	s	8.4437
Fumarate	CH	s	6.5087
Galactose	CH	d	5.2610
Glucose	CH	d	4.6320
Glucose-1-phosphate	CH	dd	5.4601
Glutamate	CH_2-4	m	2.3363
Glycine	CH_2	s	3.5530
Hippurate	CH-3	t	7.5564
Histidine	CH	s	8.0919
Isobutyrate	CH_3	d	1.0615
Isoleucine	CH_3-4	d	1.0007
Lactate	CH_3	d	1.3222

Lactose	CH-2	t	3.2869
Leucine	CH_3	t	0.9464
Lysine	CH_2-6	t	3.0150
Malonate	CH_2	s	3.1238
Maltose	CH	d	5.4001
N,N-Dimethylglycine	CH_3	s	2.9039
N-Acetylglucosamine	CH_3	s	2.0436
O-Acetylcarnitine	CH_3	s	3.1781
Phenylalanine	CH-6	t	7.4142
Pimelate	CH_2-2	t	2.1449
Proline	CH_2-5	m	3.3509
Propionate	CH_3	t	1.0540
Propylene glycol	CH_3	d	1.1212
Pyruvate	CH_3	s	2.3616
sn-Glycero-3-phosphocholine	CH_3	s	3.2147
Succinate	CH_2	s	2.3949
trans-Aconitate	CH-2	s	6.5782
Trimethylamine N-oxide	CH_3	s	3.2593
Tyrosine	CH-6	d	6.9021
Uracil	CH-2	d	5.7917
Uridine	CH-4	t	5.9087
Valine	CH_3	d	0.9770
Xylose	CH	d	4.5790

S stands for singlet, d stands for doublet, t stands for triblet and m stands for multiple signals.
